# What Supports and Constrains the Implementation of Robust and Rapid Evaluation Within Local Government Public Health Systems?

**DOI:** 10.1111/hex.70650

**Published:** 2026-03-26

**Authors:** Gemma Bridge, Jane Wills, Jessica T. Oha, Thomas Mills, Apolonia Radomska, Susie Sykes

**Affiliations:** ^1^ College of Health and Life Sciences, School of Applied and Health Sciences London South Bank University London UK

**Keywords:** co‐production, local government, public health, rapid evaluation, systems

## Abstract

**Background:**

Since 2013, local government in England has held statutory responsibility for public health and concurrently faced financial pressures and limited research capacity. The National Institute of Health and Care Research (NIHR) established Public Health Intervention Responsive Studies Teams (PHIRST) to bridge this gap, commissioning rapid, co‐produced evaluations of complex interventions.

**Main Body:**

Drawing on 5 years of PHIRST South Bank experience and ten diverse evaluations, this paper explores what supports and constrains collaborative evaluation in politically responsive, resource‐constrained local systems. Learnings from across evaluations include the dilemmas of balancing timeliness and rigour, the centrality of relationships and relational infrastructure, and the importance of building research and evaluation capacity in local government teams from the outset. We also consider the applicability and limits of existing evaluation frameworks for system‐level interventions.

**Conclusion:**

We argue that collaborative evaluation is both necessary and fragile: dependant on community engagement, adaptive design, balancing of demands, and building of intersectoral relationships.

**Abbreviations**
HDRC: Health Determinants Research Collaborations
LA: local authority
LARP: local authority research practitioner
MRC: Medical Research Council
NHS: National Health Service
NIHR: National Institute of Health and Care Research
PHIRST: Public Health Intervention Responsive Studies Teams
PPIE: Patient and Public Involvement and Engagement
VCFSE: Voluntary, Community, Faith and Social EnterprIse

## 1 Background

In 2013, responsibility for public health research and evaluation in England transferred from the National Health Service (NHS) to local authorities (LA), under the Health and Social Care Act 2012 [1]. Prior to 2013, most public health activity in England was situated within the NHS, where evaluation was more commonly embedded within established health service research structures, with links out to university‐based research expertise if and when needed. The transfer of statutory public health responsibilities to LA broadened the scope of action on the social determinants of health, enabling closer alignment with sectors such as housing, transport, planning, and community services. However, it also relocated public health into politically responsive and resource‐constrained settings, often without equivalent research infrastructure [2, 3].

As a result, while LA's were well positioned to design and deliver place‐based interventions, many faced practical challenges in generating timely and robust evidence about their impact. Initiatives such as the National Institute for Health and Care Research (NIHR) Public Health Intervention Responsive Studies Teams (PHIRST) programme were established in part to address this gap. PHIRST teams offered rapid evaluations to ease pressures [4]. Such evaluations supported both rapid decision‐making and the longer‐term evidence base, and benefit both local government and researchers [5]. Yet rapid evaluation also poses dilemmas around rigour, depth and comparability [6].

While a growing body of literature discusses evaluation of complex public health interventions and systems‐oriented approaches, less attention has been paid to how such evaluations are implemented in practice within LA settings. There is also limited empirical reflection on how collaborative rapid evaluation models function in real‐world LA systems and what enables or constrains their implementation. Drawing on the first 5 years of PHIRST South Bank's experience across 10 evaluations, this paper examines what supports and constrains the implementation of robust and rapid evaluation within local government public health systems.

## 2 PHIRST South Bank: 5 Years of LA Evaluation Experience

In its first 5 years (2020–2025), PHIRST South Bank undertook 10 rapid evaluations (with a typical time frame of 16–18 months) of interventions delivered by LAs (and/or their partners) across England (see Table 1).

**Table 1:** Summary table of the 10 evaluations conducted by PHIRST South Bank between 2020–2025.

Evaluation title and dates	The intervention	The evaluation	Findings
A realist evaluation of a public health community of practice advocacy project to restrict outdoor advertising of high fat, salt, and sugar (HFSS) foods. Sept 2020–May 2022	A Healthy Weight and Physical Activity Community of Improvement have been working since June 2020 to lead a public health advocacy project across an English region. The intervention aimed to deliver a regional approach to the development and implementation of local policies to support the reduction of advertising of HFSS products.	The aim of the evaluation was to find out what makes it easier or more difficult for local government public health professionals and others to influence council policy on the advertising of HFSS products. A mixed‐methods realist evaluation was carried out to assess progress and impact in restricting advertising of HFSS products.	Public health advocates gained confidence through peer support, clear messaging, and political backing. Advocacy was slow and resource‐intensive, requiring strong leadership and sustained effort. A phased stakeholder engagement approach helped build support but caused delays and tensions. The Policy Advocate role was vital yet demanding, often stretching skills and capacity. Advocacy was recognised as a distinct public health function, supported by regional coordination and expert input.
Evaluation of the impact of Essex Community Action Site upon Attitudes, Behaviour and public health Systems during the COVID‐19 pandemic. Essex January 2021–March 2022	Essex Coronavirus Action Support (ECAS) involved collaboration between Essex County Council (ECC) and the Essex Public Health Team as well as local Facebook groups. The intervention focused on three objectives: preventing the spread of infection, informing Essex residents of guidance and assisting residents who may be vulnerable.	The evaluation investigated project outcomes and contributing factors for improved health literacy, protective health action, community connectedness and mutual aid, and the extent to which whole system change was achieved for the public health function. Employed a range methodologies including primary analyses of survey data, social network analysis, discourse, and sentiment analysis of Facebook group interactions, alongside interviews with key stakeholders.	Social network interventions can act as dependable information sources and may be particularly helpful for those feeling socially isolated. The intervention provided a space for people to maintain social contact whilst addressing confusion and concern. Disseminating public health guidance via this new means allowed a two‐way interaction, differing from traditional forms of media. Participants in the evaluation hoped that the intervention would lead to empowerment within communities and may play a part in system change. The intervention showed promise for expanding the reach of public health messages for societal benefit.
Evaluation of the development of a behaviour change unit (BCU) and its contribution to local government. March 2021– December 2022	The BCU sits within the Public Health directorate, has a Council wide remit and works with external partners. It aims to embed behavioural science in a broad sense across the council, encourage the adoption of ‘behavioural thinking’ and support the use of evidence and tools across directorates.	The evaluation aimed to determine what are the short‐ and medium‐term outcomes of the BCU and how have these been achieved. A convergent parallel design was used with elements conducted sequentially to inform subsequent stages. The evaluation included: Documentary analysis Interviews with senior managers, a questionnaire staff identified behavioural science champions and focus groups with staff from directorates.	The evaluation found that a clearly defined and well‐communicated remit would help staff engage with the unit and enable leadership to champion its value. The BCU service's role was unclear to some and ranged from offering communication advice to acting as project managers. Staff were more supportive when they understood the purpose and saw how the intervention aligned with broader goals such as equity, prevention, or public health priorities like increasing physical activity.
Greater Manchester Communities Addressing Gambling Harms September 2021–April 2023	The Greater Manchester Gambling Harms (GMCA) Communities of Practice (CoP) was set up to facilitate regional action on Gambling Related Harm (GRH). The GMCA CoP that brings together people with Lived Experience of GRH and diverse community organisations.	The evaluation aimed to develop a Theory of Change for a Community of Practice of community organisations and Lived Experience personnel seeking to facilitate regional action on GRH. A mixed methods approach was deployed with an emphasis on qualitative methods to explore the process‐oriented research question and objectives. A short impact survey of internal and external stakeholders was also undertaken at the endpoint: external stakeholders were those deemed, by community project staff, to have a unique perspective on impact.	‘Communities Addressing Gambling Harms’ effectively fostered innovation in community‐based GRH reduction. The strongest evidence of impact was on public awareness, likely enhanced by commissioning multiple community projects together. The CoP promoted learning on gambling narratives, collaboration across projects, and cross‐referrals, evolving into a regional GRH reduction network with an integrated support pathway. Lived experience contributors played a major role, combining formal and personal efforts to support others and drive social change.
Evaluation of a public health pathway for alcohol and substance users in the criminal justice setting in Nottinghamshire Sept 2021–May 2023	The intervention included a public health pathway for alcohol and substance users in the criminal justice (CJ) setting in Nottinghamshire. The model proposed consists of a new referral process within the custody suite that will allow for a proactive, targeted and layered approach for addressing the needs of different cohorts in Nottinghamshire.	The evaluation aimed to generate policy and service recommendations for CJ substance abuse services through a robust, mixed‐methods evaluation of an innovative pilot. The evaluation employed a mixed methods approach including observations, document analysis of national policy documents and local service documentation, semi‐structured interviews with senior staff stakeholders, staff potentially involved in delivering, referring into or working with service‐users along the pathway and core CGL and PH Commissioning staff.	The intervention experienced significant implementation barriers due to time pressures, staff turnover, and limited skills or confidence among custody police to deliver enhanced assessments. Substance misuse workers were seen as vital but scarce resources, with gaps in substance misuse skills and knowledge among police and NHS Liaison & Diversion staff. Both custody and community settings offered opportunities and challenges‐ custody provided established processes and a ‘golden moment’ for engagement, while community routes supported upstream prevention but lacked established pathways. Processes for identifying and managing problematic drinking in custody were unclear, leading to missed opportunities for prevention and highlighting the need for clearer protocols and training.
The change from face‐to‐face to telephone specialist smoking cessation service (SSS) and its effects on underserved communities in Bedford, Central Bedfordshire and Milton Keynes November 2021–February 2024	Smoking Cessation Service Redesign: In transitioning to a hybrid model of service delivery, combining face‐to‐face and telephone support	The aim was to generate policy and service recommendations for councils across England through a robust, mixed‐methods evaluation of an SSS that has shifted to telephone delivery Employed a mixed methods approach – looked at data to compare quit rates between telephone delivery and face to face sessions; utilised economic methods to assess difference in cost between phone and face to face delivery. Surveys, interviews and focus groups captured insights into how people perceive the programme.	The evaluation highlighted how attention to equity was built in from the outset. The service adapted its offer based on population need, ensuring digital access was supported rather than assumed, and that high‐need groups retained access to in‐person support. This intentional design helped prevent widening inequalities while expanding reach.
Reducing inequalities in exercise participation: Evaluation of the novel ‘Bracknell Forest Health and Well‐Being Locality Service’ (BFHWBS) April 2022–March 2024	The intervention aimed to encourage physical activity to improve health and quality of life for residents. The service is free of charge for those from socially disadvantaged communities. People can also self‐refer to the service or be referred by a social prescriber instead of having to be referred by a General Practitioner.	The evaluation aimed to evaluate how the novel aspects of the service help to reduce inequalities in physical activity participation. The evaluation investigated whether the structures and agents in the local system effectively supported physical activity delivery. The evaluation employed a mixed‐methods approach including a systems mapping, qualitative and statistical service user data, a cross‐sectional street intercept face‐to‐face survey, and a small‐scale descriptives cost analysis.	Rather than positioning the exercise referral service as a standalone intervention, the evaluation showed the importance of embedding it within a wider locality‐based system of health and wellbeing and ensuring it connected with primary care, social prescribing, community development, and neighbourhood teams ‐ creating a more joined‐up, place‐based model.
A mixed methods evaluation of a complex intervention: Improving the mental health of 16‐25‐year‐olds in Central Bedfordshire January 2023–September 2024	The intervention in Central Bedfordshire aimed to improve the mental health of young people aged 16–25, particularly those most in need such as ethnic minorities, LGBTQ+ individuals, and young carers. Uniquely, the evaluation combined Population Health Management (PHM) and Community Collaborations (CC) approaches to enhance access, quality, and coordination of mental health support.	This evaluation sought to understand the impact of a complex mental health intervention targeting young people. It looked to unpack the process and experience of implementing PHM and CC approaches towards improving mental health outcomes for young people groups most in need (incl. ethnic minorities, LGBTQ+ individuals, and young carers). The evaluation employed mixed‐methods analysis to explore programme effectiveness, implementation processes, involvement and engagement and exploration of service user experiences.	The intervention was partially effective in addressing young people's mental health and wellbeing, with short‐ and interim‐term outcomes partly achieved and long‐term outcomes likely if funding continued. Significant adjustments were needed to fully deliver a Population Mental Health (PMH) approach supporting sustained outcomes. The Population Health Management (PHM) approach aided outcome delivery, particularly through data integration, though gaps remained in linking social care, health data, and wider determinants. Community Collaborations highlighted the importance of relationships, accessibility, proportionality, reduced focus on diagnosis, and engaging delivery spaces for future success.
A process evaluation of the development and early implementation of a complex strategy to reduce serious youth violence in Lambeth April 23–April 25	A complex 10‐year strategy (Lambeth Made Safer, LMS) aimed to reduce serious youth violence in Lambeth, London ‐ a strategy level public health approach.	The evaluation aimed to understand what shaped strategy development and the wider contexts in which the strategy operates; to explore the lived experiences of those involved in strategy interventions; to identify opportunities for strengthening measurement of strategy outcomes; to understand strategy costs and consequences from a public perspective. The evaluation conducted a process evaluation (parallel mixed methods design) on the development and early implementation of the LMS	The evaluation highlighted the importance of coordinated multi‐agency work in building a shared understanding of youth violence as a public health issue – not just a criminal justice one. Also highlighted range of enablers for successful LMS delivery as well as challenges, including Individual, relational, community, and societal factors. The evaluation was highly collaborative, with co‐production at the core of the evaluation. Success of the evaluation hinged on collaboration between many actors across the public and voluntary sectors. Involved young people and carers/parents in the evaluation.
Evaluation of how ‘hybrid’ public health posts in local government support the delivery of healthier places in the Oxford‐Cambridge Arc January 24–July 25	Local authorities are increasingly encouraged to combine expertise in planning and public health and to create local'places’ that promote people's health. Some public health teams are now creating dedicated posts and teams to work alongside their colleagues in planning. This initiative involved placing public health specialists at the interface with local government planning teams.	The evaluation aimed to generate insight into the activities and impact of public health posts for supporting the delivery of healthier places. The study used a mixed qualitative approach across four work packages (WPs). WP1 involved desk‐based research, stakeholder interviews, and meetings with external stakeholders to develop a framework for delivering healthier places. WP2 used action mapping workshops, postholder diaries, and interviews to understand the posts from core team perspectives. WP3 gathered insights from interviews, focus groups, and analysis of local plans to assess local stakeholder views and impacts. WP4 analyses resource use, budget data, and qualitative findings to explore how resources were used and the benefits generated.	The evaluation found that flexibility allowed each authority to shape the role to local needs and organisational structures, making the intervention more effective and more likely to be sustained. In terms of the local plans – potential implementation challenges in development management due to competing priorities, skill gaps and workforce pressures. Timely and bespoke support from ‘hybrid’ postholders as a key enabler for both place making and implementation.

The evaluations spanned an array of public health issues, such as the creation of hybrid public health posts within planning teams [7], the implementation of advertising restrictions on high fat, high sugar food and drink [8], community‐led responses to gambling‐related harms [9], and the evaluation of a strategic approach to serious youth violence [10]. Together, these cases illustrate both the breadth of LA responsibility for the social determinants of health and the opportunities they offer for developing varied evaluative approaches.

### 2.1 Lessons From Across the 10 Evaluations

This paper draws on a reflective cross‐case synthesis of the ten evaluations conducted by the PHIRST South Bank team between 2020 and 2025. The evaluations used a range of methodological approaches (including realist‐informed, process, and mixed‐methods designs) appropriate to the interventions being studied. For this paper, insights were generated through team‐based reflection across the evaluations, reviewing evaluation reports, fieldnotes, and outputs to identify recurring methodological, organisational, and relational themes shaping the implementation of collaborative rapid evaluation in LA contexts. The analysis therefore focuses on cross‐cutting lessons rather than comparative effectiveness of individual interventions and highlight how interventions are rarely self‐contained programmes but instead are embedded in complex systems shaped by political cycles, organisational cultures, and community dynamics. These reflections add to those of other rapid evaluation teams, highlighting the importance of sharing lessons more effectively [6, 11].

#### 2.1.1 Embedded in Complex Systems

The interventions evaluated by the PHIRST South Bank team were embedded in complex local systems shaped by political cycles, organisational cultures, and community dynamics [12]. Evaluations thus required close working relationships with LA teams, representatives from voluntary, community, faith and social enterprise (VCFSE) sectors, and members of the public, as well as attention to connectivity, context, and contribution. UK Medical Research Council's (MRC) 2021 guidance has been an anchor point across the PHIRST South Bank evaluations [15].

The evaluation of health and wellbeing physical activity service delivered by an LA in central England was an example of working within this complexity [13]. The evaluation required the involvement and engagement of many agencies and structures to capture, and assess uptake, engagement, acceptability and impact of the intervention amongst users and target populations. The evaluation revealed that intervention effectiveness depended not only on the service itself, but on its integration with primary care, social prescribing, community development, and neighbourhood teams, underscoring that system connectivity is often as important as service design in complex systems.

Similarly, the evaluation of the development and early implementation of a complex strategy to reduce serious youth violence in a highly diverse London Borough [10] demonstrated that progress depended less on technical content and more on partnership strength across public health, policing, and community organisations. Workshops and peer researchers helped surface relational dynamics shaping implementation.

From these PHIRST South Bank experiences, we argue that evaluations conducted in complex LA settings, need to be undertaken so that they reflect not only the intervention design but also the relational infrastructure, the partnerships, trust, and negotiation that enable delivery. In support, Van der Graaf et al. [14] recently conducted research to assess facilitators for Whole Systems Approaches in public health, and emphasised the importance of working collaboratively across sectors, building a consensus around values and developing mutual awareness of roles and structures. Further to this, recent updates to the MRC guidance on complex interventions [15] advocated for systems orientated, and participatory approaches that can capture unexpected and non‐linear change and outcomes.

However, such approaches can be time and resource demanding and carry practical challenges. Potential participants in LAs, public health and social policy teams often have limited time to engage in evaluations and are constrained by fragmented data [16].

#### 2.1.2 Balance Between Timeliness and Rigour

LA teams require evaluation findings within short time frames to inform service and commissioning decisions, and/or policy cycles. However, limited data, and ad hoc communication between researchers and LA teams can risk limiting opportunities for discussion, joint sense‐making. Tension in time, communication and expectation can manifest as breadth versus depth, flexibility versus fidelity, and formative learning versus summative judgement.

Experience gained over the first 5 years of PHIRST South Bank has shown that it is possible to work rapidly, whilst maintaining rigour and delivering key insights to tight LA informed deadlines. For example, by using scoping and realism of convergent parallel designs, staged reporting, and real world co‐produced logic modelling to keep focus tight. Pre‐agreed ‘claims limits’, making explicit what evaluators can and cannot investigate, have also helped the PHIRST South Bank manage expectations. Further to this, is the embedding of mechanisms for ongoing dialogue, interim reporting and regular communications, rather than relying on lengthy end‐of‐project reports.

The craft lies in aligning the level of rigour with the intended use, ensuring that findings are sufficiently robust to support sound decision‐making while avoiding unnecessary methodological escalation that would undermine timeliness. The development of STREAM (Standards for Rapid Evaluation and Appraisal Methods) [17], as well as other contributions from the Rapid Research Evaluation and Appraisal Lab (RREAL) [18], reflect increasing recognition that policymaking must prioritise timely, fit for purpose evidence.

The evaluation of a specialist smoking cessation service that changed from face‐to‐face to a telephone ‘quit line’ delivery [19] illustrates the balance of matching rigour to use. A mixed‐methods design enabled timely analysis of access, uptake, and early quit outcomes. The participatory co‐inquiry approach, conducted across six facilitated sessions, supported real time reflection and led to changes in how the LA undertook community outreach [20].

However, as has been the case with most of the evaluations that the PHIRST South Bank team have worked on, and reflecting the pressured and resource poor environments in which LAs work, the routine service data available in the evaluation of the specialist smoking cessation service were incomplete and of variable quality, making it challenging to conduct robust comparative analyses between telephone and in‐person delivery, and assessing impacts of different community groups.

Despite the challenges, the evaluation provided an important learning opportunity for the PHIRST South Bank team, and for the LA team: *‘We realised we weren't collecting the right data or storing the right data to evaluate our services. So we made that change and we are in the process of developing a new data system for a similar services’ ‐ LA staff member, Stop Smoking evaluation*

The evaluation of the hybrid public health and planning posts in the Oxford–Cambridge Arc highlighted a different dimension of the timeliness‐rigour dilemma [21]. Early findings from the evaluation documented strengthened relationships, improved visibility of health considerations in planning, and new models of cross‐departmental working. However, it was not possible to assess population level or long‐term planning impacts, and before the evaluation could make a robust case for sustaining the roles, one of the teams was decommissioned. This underscores a recurrent dilemma for local government whereby decisions about continuation or cessation of interventions (such as innovative posts) may be made before evaluative evidence is available, privileging immediate outputs over longer term change.

A promising recent development is the establishment of NIHR Health Determinants Research Collaborations (HDRCs) which are enabling some LAs to generate, use, and share evidence as part of their routine decision‐making [22].

#### 2.1.3 Partnerships, Co‐Production and Relational Infrastructure

Trust between evaluation teams, LAs, and intervention delivery teams, supports access to data and insights, and adaptation to evaluation design, whilst co‐production and an awareness of on the ground need and interests helps to align evaluation questions with policy and practice windows. In the experience of PHIRST South Bank, strong relationships between the evaluation team, LAs and intervention delivery teams helps generate findings that are well understood, valued and used by LA partners.

In reflection of the importance of collaborative working, all evaluations conducted by PHIRST South Bank involve working closely with LAs, delivery partners, as well as with people from the local communities where interventions take place. PHIRST evaluations also often includes those with lived experience of the public health issue under study. Community members are engaged through advisory groups and locally recruited patient and public involvement and engagement (PPIE) panels (acting as public contributors), rather than relying solely on centrally managed, or pre‐existing PPIE structures.

Across the evaluations PPIE panels shaped scoping, data tools, and interpretation. For example, in the evaluation of community‐led responses to gambling‐related harms [23], people with lived experience shaped evaluation questions, and supported with understanding wider system, and the evolving dynamics of partnership and influence. In the evaluation of the Serious Youth Violence Strategy, peer researchers strengthened relevance and trust.

Embedding co‐production – working with public contributors and working closely with LA teams ‐ from the outset of evaluation is not simply about inclusivity as a principle, but also about generating more relevant and actionable evidence for LA's and the communities that they exist to serve. But, co‐production is time‐intensive, and the deeper the engagement, the harder it can be to sustain in contexts where political timescales are short, and staff capacity is stretched. Further to this, some LA partners prefer a more independent evaluation, valuing the external view of an impartial research team, over close collaboration. These variations highlight the importance of clarifying roles, and the involvement of evaluators, and community groups from the outset.

#### 2.1.4 Equity and Inclusivity as Principles of Intervention Design and Evaluation

Equity is a statutory obligation within public health practice in England (Health and Social Care Act, 2012), and as such, LAs have a legal duty to reduce health inequalities for the whole population. Unsurprisingly, equity surfaced as a recurring theme across all 10 PHIRST South Bank evaluations – but the way in which equity was conceptualised and operationalised varied. Where equity had been operationalised into intervention design, scoping discussions for evaluations could more easily consider questions such as – Who might be excluded? What adaptations are needed? How will the intervention address structural barriers? In doing so, evaluations could explore mechanisms, contexts, and plausible pathways to reducing existing inequalities.

The redesigned Stop Smoking service exemplified intentional equity by design [19]. In shifting to telephone delivery, the LA team invested in support for access to a wide array of people but also recognised the potential exclusionary risks of a telephone only model. With this consideration in mind, the PHIRST South Bank evaluation team could explore reach and the conditions under which equitable outcomes could be sustained.

By contrast, in other interventions, such as the evaluation of healthy planning posts [7] and the locality‐based physical activity wellbeing service [13], were less explicit at design stage about equity, therefore attributing equity impacts proved was more difficult within evaluation short timescales. Compounding this, is the impact of the wider, complex system, where equity impacts may unfold over years, beyond the scope of rapid evaluation.

When evaluating LA interventions, equating equity with reach or demographic coverage can mask deeper, underlying disparities. PHIRST South Bank evaluations have highlighted that equity depends not only on who takes part in interventions, but also on whether the intervention's underlying theory of change, and the activities it offers, addresses the social determinants that shape people's ability to benefit. For example, an intervention designed to increase physical activity by providing free access to local leisure centres may appear to reach all groups, yet fail to engage with barriers linked to income, mental health, cultural norms, or access to transport. Across our evaluations, structural features such as who has voice in decision‐making related to interventions, whether services are meaningfully adapted to local cultural contexts, and how communities are framed as ‘at risk’ could reinforce exclusion even when uptake looked balanced. These findings align with wider public health concerns: unless equity is embedded in both intervention design and mechanisms of change, apparently fair reach can conceal persistent and patterned inequities.

A recurring methodological dilemma, especially in rapid evaluations, is determining what evidentiary threshold is required before scaling an intervention that initial indicates is benefiting target groups but there is not long‐term outcome data to support this. In such cases, the use of theory‐driven approaches such as logic models or theories of change can be important. Such frameworks and models can help to depict, and articulate the causal pathways between activities, intermediate outcomes, and eventual equity impacts, and can be used to show, and assess how well these links are supported by existing evidence. They also make explicit the assumptions underpinning equity claims, creating a transparent basis for assessing whether early signals of impact are likely to translate into longer term, policy and/or system‐level change.

Applying established co‐production principles, such as sharing power, including all relevant perspectives, and reciprocity [24], has been an important evaluation design element for PHIRST South Bank in ensuring that evaluation processes themselves did not reproduce inequalities.

#### 2.1.5 Build Capacity and Confidence

In LA settings, staff research and evaluation literacy is pivotal to effective intervention evaluation. However, recent research suggests that many LAs lack time and resources to support practitioner research capacity and capability [25]. Challenges also exit with LA research culture more broadly for example in relation to data sharing and governance, resource pressures, siloed working, and staff turnover. As a result, evaluators working with LA teams often rely on the engagement of individual staff members or small teams with an interest in research ‐ making efforts fragile and uneven. Evaluative partnerships can therefore be vulnerable, underscoring the need for more systemic capacity‐building strategies.

There have been efforts made to support LAs to undertake research and evaluation in house. Research support service (RSS) specialist centres for public health were launched to support the development of research capacity and capability – giving LA teams, as well as other researchers working outside the NHS – access to a wide range of public health expertise from skilled methodologists and public health experts. Some LAs also now have embedded researchers (Local Authority Research Practitioners, LARPs), employed as part of HDRCs, who can offer dedicated time and resources to support evaluation. However, LARP posts are not available across all LAs, nor do they span all topic and intervention areas.

Rapid collaborative evaluations, such as those carried out by the PHIRST centres, can work alongside RSS centres, LARPs and wider HDRC teams to build LA evaluative capacities, helping to validate practice, build research skills, and enhance confidence in LA staff [26], and PPIE panels – as exemplified by one PPIE contributor, ‘I learnt so much, and it gave me so much more opportunity in the work I am doing now’.

LA staff also stated how the additional support from the PHIRST South Bank team provided them with the time and ‘head space to reflect…’ – important for developing, planning and carrying out evaluations. For example, in the evaluation of the Gambling Harms CoP, due to the politically contentious policy area in which the gambling industry can be hostile, staff from the LA and community partners needed support and a safe space in which to discuss the issues and start to address the problem. In support, LA staff emphasised the usefulness of having additional, external research capacity to ‘think about what we are doing, why we are doing it and how are we going to get there [the outcomes] and start to design what we are doing to what around what we were trying to achieve’.

In support, in the realist evaluation of the high fat, high sugar (HFSS) advertising restrictions [27], the evaluation offered a mechanism for building the confidence and capacity of local actors to effect change. Practitioners initially reported limited confidence in advocating for policy change, but over time, through peer support, clear messaging, and political endorsement, reinforced by evaluation findings, the practitioners were able to build the relevant skills and gain improved legitimacy.

## 3 Engaging With Evaluation Theory, Frameworks and Methods

Prior to the 2013 transfer of public health responsibilities, many evaluations were undertaken within NHS settings where established analytic teams, public health observatories, and national datasets supported routine monitoring and service evaluation. By contrast, the examples presented in this section reflect evaluations conducted within LA systems operating under sustained fiscal constraint and organisational change. In these settings, evaluation must often be conducted alongside delivery, with limited analytic capacity, fragmented data systems, and competing political and operational pressures. As a result, the examples illustrate not only the application of frameworks or methodological approaches, but also how evaluative thinking is adapted to the realities of local government decision‐making and resource constraints. The examples also reflect principles associated with learning health systems, where data, evaluation, and stakeholder dialogue are used iteratively to inform ongoing improvement and decision‐making within complex service systems.

PHIRST South Bank evaluations to date have been guided by the Medical Research Council (MRC) Framework for Complex Interventions [15], which emphasises designing theory‐based, flexible, and culturally sensitive evaluations capable of capturing systems dynamics and adaptation processes. This approach promotes understanding of how interventions work within complex systems, focusing on mechanisms, contexts, and outcomes, aligning well with the multi‐faceted realities faced in LA settings. Complementing this, the Magenta Book guidance [28] provided principles for proportionate evaluation, emphasising early planning, methodological flexibility appropriate to intervention scale, and the continuous use of evaluation for improvement and accountability. Furthermore, the evaluations incorporated principles from implementation science such as fidelity, acceptability, sustainability, and systems thinking [29]. This reinforced the importance of understanding how interventions are experienced, adapted, and embedded within local systems.

### 3.1 Understanding Context: Complexity, Systems, and Evaluative Thinking

Working in LA settings requires what Vo and colleagues [30, 31] term evaluative thinking ‐ a mindset oriented to uncertainty, adaptation, and system interdependencies. Evaluative thinking has been important across all PHIRST South Bank evaluations – helping the team to attend to how shifting contextual pressures and dynamics shaped what is feasible.

For example, in the evaluation of a new pathway concept for alcohol and substance users in the criminal justice setting in Nottinghamshire, we found that whilst there was an increase in funding for the pathway concept from the National Government, custody police staff lacked the time, confidence and skill to undertake enhanced assessments of service users, limiting implementation effectiveness, and there was high staff rotation complicating efforts to train and motivate staff, and limiting efforts to engage with staff during the evaluation.

To operationalise contextual complexity, PHIRST South Bank evaluators have used systems‐oriented tools. For example, utilising stakeholder mapping and real‐world logic modelling during scoping exercises and employing techniques such as social network analysis in data collection. Such approaches have helped to surface assumptions, visualise interacting influences, and anticipate adaptive pressures which support a more realistic basis and understanding of interventions for evaluation design in complex, and changing LA environments [32].

### 3.2 Understanding Assumptions About How Activities Lead to Outcomes: Programme Theory, Logic Modelling and Theories of Change

Interventions in LA settings are rarely discrete or standardised, and as such, programme theory is an important step in designing evaluations. In PHIRST South Bank evaluations, early workshops with LA partners use basic logic models to map intended pathways of change, which can then be refined iteratively to capture adaptations, feedback loops, and interactions with wider systems. These logic models support a shared language between practitioners, evaluators, and community members, strengthening both design and understanding.

For example, in the mixed methods evaluation of a complex intervention to improve the mental health of 16–25‐year‐olds [33], developing a theory of change with practitioners and young people helped make explicit the intended relational mechanisms (trust, reduced stigma, help‐seeking confidence), and provided a structured way to understand how the intervention's components – including the four intervention streams – led to observed outcomes. We have also employed more dynamic and flexible logic model designs [34, 35] to enable teams to creatively theorise interventions within the context of their delivery.

### 3.3 Approaches Suited to Complex Systems: Realist, Developmental and Systems‐Based Evaluation

#### 3.3.1 Realist Evaluation

Realist evaluation [36] encourages systematic inquiry into context–mechanism–outcome (CMO) configurations, which has been useful for evaluations of interventions embedded in multi‐agency systems. Realist reasoning examines what works, for whom, in what contexts, and why. Such an approach has been used in PHIRST evaluations over the last 5 years.

This was explicit in the PHIRST South Bank evaluation of a public health community of practice advocacy project to restrict outdoor advertising of high fat, salt, and sugar foods [27] on council owned spaces. A realist lens helped the team understand how public health advocacy mechanisms interacted with political conditions, commercial pressures, and practitioner confidence to influence policy outcomes.

Realist evaluation is particularly valuable in public health evaluation because it seeks to understand how context interacts with mechanisms to produce outcomes, recognising that interventions operate across multiple societal structures, including policy environments, organisational settings, and community contexts. However, fully operationalising realist evaluation is often constrained by rapid timelines, the availability of routine data, and the practical limits of practitioner and community capacity. Thus, ‘realist‐informed’ rather than fully realist designs have been used, balancing theoretical integrity with pragmatism. This flexible approach to realist design has been discussed as a consideration of realist approaches in other fields [37].

#### 3.3.2 Developmental Evaluation

Developmental evaluation is relevant where interventions are responsive and adaptive [38]. In this context, LA partners value evaluators working alongside them to support iterative adaptation rather than delivering summative judgements. For example, as part of the evaluation of the Stop Smoking Service transition from a face‐to‐face to telephone service, a co‐inquiry approach was adopted to formatively evaluate staff's community outreach. This approach involved staff collecting data, and then reflecting on this with the evaluation team via facilitated six reflection sessions over a 12‐month period – to draw out implications for practice and the real‐world logic model being developed. The co‐inquiry approach was participatory and supported learning and service improvement which supported shared understanding and refinement of outreach efforts by LA staff.

Developmental approaches, however, require continuity of relationships, tolerance for uncertainty, and protected time for reflection ‐ all of which are challenging in LA environments affected by austerity, high staff turnover, and short‐term political cycles. Developmental evaluation can produce insights, that whilst valuable for specific regions and relationships, can be harder to capture and report back to funders, wanting evidence of impacts.

#### 3.3.3 Systems‐Based Evaluation

Many PHIRST South Bank evaluations were system‐shaping rather than programme‐specific. For example, this was important in the Health and Wellbeing Physical Activity Locality Service evaluation, where the evaluation needed to understand the service not as a standalone offer but as part of a wider ecosystem involving primary care, social prescribing, and community development. Mapping system connections identified hidden bottlenecks and opportunities for strengthening referral pathways, intermediate outcomes that would have been missed by narrow measurement frameworks.

Yet, applying whole‐systems methods in practice can be hindered by fragmented datasets, limited time for participation (Riley et al., 2024), and challenges in attributing outcomes within adaptive systems (Whitelaw et al., 2025). Moreover, whilst evaluations aim to provide insights that can support decision making, doing so is challenging when the interventions themselves are complex (made up of multiple components and with many different people involved), the environment is complex (made up of multiple organisations and rules that change regularly), and the impacts of the interventions are also complex (with different impacts felt by different people at different time) [39].

The PHIRST evaluation examples illustrate how evaluation frameworks are interpreted and adapted in contemporary LA contexts. Unlike pre‐2013 public health evaluators that were embedded within the NHS, evaluators now work within systems where analytic capacity is uneven, interventions evolve rapidly, and policy priorities may shift in response to political and financial pressures. Under conditions of austerity, the challenge for evaluators is organisational as well as methodological whereby evaluation must be sufficiently pragmatic to inform local decision‐making while maintaining credibility and analytical rigour. The cases discussed above demonstrate how evaluators can draw from, and combine multiple traditions, such as realist, developmental, and systems‐informed approaches, to generate useful insights within constrained and adaptive policy environments.

## 4 The Future of Collaborative Rapid Evaluation in LA Settings

The reflections from the PHIRST South Bank team point to both the promise and fragility of collaborative evaluation in local government. Looking ahead, the question is not only how evaluations are designed, but how the conditions that support their uptake and sustainability can be created and maintained.

### 4.1 What Conditions Support Evaluation in Local Systems?

The most effective evaluations we observed emerged where there was relational infrastructure with trusted partnerships, established channels of communication, and shared language between evaluators and LA teams. These relationships made it possible to align evaluation questions with live decision‐making cycles, to navigate inevitable tensions, and to generate findings that were both used and useful.

Beyond relationships, three further conditions stood out, namely organisational leadership that valued evidence; space for staff to engage meaningfully with evaluation amidst competing priorities; and meaningful engagement and involvement of communities and people with lived experience. Literature on evaluation capacity building (e.g.) [26, 40] has similarly highlighted the importance of leadership, learning culture, and resources as critical enablers. The PHIRST South Bank experience suggests that without such foundations, even well‐designed evaluations risk becoming isolated exercises.

Frameworks such as the Theoretical Domains Framework (TDF), and the Consolidated Framework for Implementation research (CFIR) provide support for, and structured ways of undertaking evaluations in LA setting by examining organisational behaviour, implementation contexts, and determinants of change. Whilst other guidance for evaluation such as the HM Treasury Magenta Book (2020) and the Government Evaluation Task Force guidance support evaluation planning, considerations of proportionality and accountability.

### 4.2 How Can Evaluation Contribute to Democratically Legitimate, Equity‐Focused Local Government?

LAs in England are elected bodies, tasked with stewarding public resources and shaping the determinants of health in ways that reflect community priorities. Evaluation therefore contributes directly to democratic legitimacy. It does this by providing an evidentiary basis for decisions about the health and wellbeing of communities, enables accountability to citizens and communities, and opens space for deliberation about what counts as success [41].

There are national repositories of evidence on interventions and programmes, such as What Works Centres – which capture evidence to improve the design and delivery of public services [42], and The Evaluation Task Force – which aims to ensure evidence and evaluation sits at the heart of spending decisions such as those related to public health [43]. However, the resources and outputs available rarely capture local nuance, and as such effort and funding are needed to enable and capture local evaluation and learning also – as is done through the PHIRST centres.

Yet, as the body of locally generated evidence grows through programmes such as PHIRST, there is an opportunity to move beyond isolated case studies toward synthesis and shared learning. Comparative analyses across evaluations could identify recurring mechanisms, transferable lessons, and contextual factors shaping implementation success or areas for further learning. Investing in the opportunity to share localised insights, linking local specificity with system‐level insight, could strengthen both national evidence infrastructures – such as those developed through the What Works Centres and The Evaluation Task Force, and the context specific value of evaluation itself.

The inclusion of public contributors and people with lived experience across the PHIRST South Bank evaluations is also a critical aspect of local evaluations moving forward, demonstrating how evaluation can be an instrument for equity and voice as well as for technical judgement [44]. However, the process of working together to achieve an equity focussed local government is not without challenge. As others have noted [45, 46], co‐production within evaluation is marked by tensions around power‐sharing. Evaluators hold authority over analytic framing and interpretation, while local partners control access to data, resources, and legitimacy. In this sense, evaluation in local government can be seen as a ‘tug of war’ (Warwick‐Booth et al., 2024), with power shifting between evaluators (LA partners and intervention leads), the evaluated and communities at different stages of the process. These dynamics are intensified in political LA environments, where findings can have implications on the sustainability of the intervention, as well as reputational, financial, or electoral implications for local government.

For PHIRST South Bank, navigating these dynamics required attention to process, clarifying expectations with LA partners and community members early, maintaining transparency about evaluation design and analytic decisions, and creating safe spaces for reflection and challenge within the research team, with public contributors, and across the wider LA and intervention stakeholders. Democratic legitimacy in evaluation, therefore, does not stem from co‐production alone, but instead, from how evaluative relationships are negotiated ‐ whose voices are heard, whose interests are represented, and how evidence is used in decision‐making.

Also important to consider here is how the findings from the evaluations are communicated. PHIRST South Bank developed tailored outputs for different audiences, including rapid briefings for LA teams and policy makers, brief presentations and webinars for practitioners, and accessible public‐facing infographics, animations and a film for residents and public audiences. These varied formats were made in recognition that decision‐making about public health takes place across multiple arenas such as political, intervention delivery, and community. With that, evaluation legitimacy relies both on generating evidence as well as on ensuring it is understandable, timely, and usable by interested parties, and those with power to act.

### 4.3 What Is the Role of Funders, Evaluators and Practitioners in Sustaining Capacity?

Sustaining collaborative evaluation between LA and academic teams requires action across multiple levels. Considering much national‐level investment is fragile and time‐limited, for funders, there is a need to support flexible and adaptive designs, rather than privileging narrowly defined outcomes. NIHR's support for PHIRSTs illustrates the potential of resourcing infrastructure that bridges research and practice, but such investment remains exceptional.

While not present in all LA settings, HDRCs and LARPs, represent a structural move from project‐level evaluation which is commissioned and time‐limited to organisation‐level evaluation cultures that support ongoing learning and strategic planning.

For evaluators, the role is not only to generate findings but also to build capability and confidence within local systems. This aligns with arguments in the literature on developmental evaluation [38], which emphasise evaluators as facilitators of learning.

Practitioners in LA settings, meanwhile, play a dual role where they are both users of evaluation and active contributors to its design and interpretation. Enabling them to participate fully requires recognition of their role in evaluation ‐ their time, skills, and relational work that can all be under‐resourced.

### 4.4 The Risks of Failing to Evaluate

Finally, there are risks in neglecting evaluation altogether. Without robust evaluation, local government interventions are vulnerable to what has been termed'pilotitis’. This refers to the proliferation of small‐scale projects that remain unscaled, their lessons lost (originally discussed in relation to digital health, but equally relevant in this context) [47].

Absence of evaluation also risks reinforcing inequities, as interventions may unintentionally benefit those already better served by systems. What is more, in politically responsive environments, policies, programmes and interventions that go without evaluation may be driven by short‐term cycles of visibility rather than long‐term considerations of effectiveness or equity. The consequence is inefficiency and missed opportunities to embed learning into the fabric of local democracy.

Adding to this, many practitioners and evaluators in public health are frustrated at the continuing emphasis on demonstrating narrowly defined ‘success’ of interventions and programmes through short‐term, easily measurable outcomes. Such approaches often privilege discrete, individual‐level interventions targeting small populations, rather than addressing the wider social, economic, and structural determinants of health, and the challenges of working within a complex system. This narrow framing not only distorts priorities but also constrains what counts as legitimate evidence, crowding out the kind of system‐level learning and adaptation that local government needs to achieve and sustain population health improvement.

As such, the future of collaborative evaluation, lies in treating it as a form of democratic infrastructure, necessary for accountability, equity, and adaptive learning in complex local systems. To realise this vision, evaluation cannot be treated as a bolt‐on to programme delivery, but instead, evaluation must be embedded as an ongoing practice of collective inquiry, messy, relational, and iterative, but also essential to governing for public value.

## 5 Conclusion

Across 10 PHIRST South Bank evaluations, several consistent themes emerged. These included the need to balance timeliness with methodological rigour, the central role of relationships and trust between researchers and LA partners, the importance of engaging communities and people with lived experience in shaping evaluation questions, and the challenges of conducting evaluation in systems characterised by limited data infrastructure and constrained resources.

Critically, evaluations require the creation of relational space for dialogue between researchers, practitioners, policy actors, and people with lived experience. These relationships enabled open discussion of political realities, implementation challenges, and community perspectives, which in turn shaped both the direction of the evaluations and how findings were interpreted and used. Our experience suggests that collaborative evaluation works best when time and attention are given not only to methods, but also to the relationships and discussions that enable evidence to inform decision‐making.

The reflections in Section Conclusion, 3 further highlight that no single evaluation framework is sufficient for the types of adaptive, system‐level interventions commonly implemented in local government. Approaches such as theory of change development, realist‐informed inquiry, developmental evaluation, and systems mapping each contributed to understanding how interventions evolved and interacted with wider local systems. Methodologically, this suggests the need for pragmatic, mixed‐framework approaches that combine rigour with flexibility.

From a policy perspective, our findings reinforce the importance of sustained investment in local evaluation capacity, time and space for relationship building, shared analytic infrastructure, and collaborative partnerships between local authorities, communities, and academic institutions if evidence‐informed public health action is to be strengthened in resource‐constrained systems.

Without this, evaluation risks remaining reactive and fragile; with it, it can support accountable, adaptive public health governance.

## Author Contributions

**Gemma Bridge:** conceptualisation, writing – original draft, writing – review and editing. **Susie Sykes** and **Jane Wills:** writing – review and editing, funding acquisition. **Jessica T. Oha**, **Apolonia Radomska**, and **Thomas Mills:** writing – review and editing.

## Ethics Statement

No ethical approval was required to write this perspective piece. All evaluations conducted by PHIRST South Bank received ethical approval from London South Bank University Health and Social Care Ethics Panel. Informed consent was appropriately obtained for all data collection activities either from participants themselves, and/or from parents/guardians when appropriate.

## Consent

Public contributors are involved in all evaluations, supporting with the design of the evaluations, the development of research tools, as well as the analysis and interpretation of the data.

## Conflicts of Interest

The authors declare no conflicts of interest.

## Data Availability

The data that support the findings of the PHIRST South Bank evaluations are not publicly available due to privacy or ethical restrictions, but some data may be available on request from the corresponding author. More information about the PHIRST South Bank evaluations, and all related outputs are available online at: https://phirst.nihr.ac.uk/about-phirst/phirst-southbank/.
